# Diseases of Children

**Published:** 1900-03

**Authors:** 


Diseases of Children. A manual for students and practitioners By George M. Tuttle, M. D., Attending Physician to St. Luke's Hospital, Martha Parson's Hospital for Children, and Bethesda Foundling Asylum, St. Louis. Illustrated with five plates in colors, and monochrone. 12mo, pp. 386. Philadelphia and New York: Lea Brothers & Co., 1899.

To attempt to condense into a small volume the essentials of such a subject as the diseases of children is a difficult task. The author in this case, however, has succeeded admirably. The physiology of infancy and the artificial feeding of the infant are dealt with at some length and in a satisfactory manner. The treatment of the various topics discussed is necessarily brief, yet nothing important is omitted, and for the beginner in the study of pediatrics it is an excellent work.

The mechanical part of the book, which is one of Lea's series of pocket text-books edited by Bern B. Gallaudet, leaves nothing to be desired. M. J. F.

